# International Comparison of Older Drivers and Non-Drivers for Activity Access and Quality of Life

**DOI:** 10.1093/geroni/igaa057.2593

**Published:** 2020-12-16

**Authors:** Cilla Harries, Carolyn Unsworth, Brenda Vrkljan, Anne Dickerson

**Affiliations:** 1 Kingston University and St George’s Joint Faculty, London, England, United Kingdom; 2 Central Queensland University, Melbourne, Victoria, Australia; 3 McMaster University, Hamilton, Ontario, Canada; 4 East Carolina University, Greenville, North Carolina, United States

## Abstract

Driving is crucial for many people to age in place, as it is the most convenient option, and somtimes the only option for personal transport. This international, cross-sectional, cohort study of older adults (n=246) compared drivers’ and non-drivers’ quality of life and levels of community participation. Following ethical approval, data were collected across seven countries. The EQ-5D-5L was used to measure health related quality of life and a modified version of the Participation in Activities and Places Outside the Home (ACT-OUT) was used to measure community participation (T-ACT-OUT). Drivers accessed more out-of-home activities than non-drivers, suggesting higher community participation among this group. Health related quality of life was generally high among all participants, but slightly higher for drivers (U=3186, z=-2.78, p=.005, r=0.18). These findings resonate with recent evidence, which suggests supporting older drivers to continue to drive for as long as possible provides critical access to their communities (O’Neill et al., 2019). Part of a symposium sponsored by Transportation and Aging Interest Group.

